# Psychological Distress in Australian Onshore and Offshore Immigration Detention Centres from 2014–2018

**DOI:** 10.1007/s10903-022-01335-7

**Published:** 2022-02-03

**Authors:** Ryan Essex, Erika Kalocsányiová, Peter Young, Paul McCrone

**Affiliations:** 1 Institute for Lifecourse Development, The University of Greenwich, Old Royal Naval College, Park Row, Greenwich, London, SE10 9LS UK; 2 Independent Scholar, Sydney, Australia

**Keywords:** Immigration detention, Health, Mental health, Offshore, Refugee

## Abstract

This study examines the impact of length of detention and location of detention on psychological distress amongst Australian immigration detainees. This study employs a repeated measures cross-sectional study, utilising Australian government data from 2014 to 2018 that relied on the Kessler-10 (K10) to measure psychological distress. There were 21,703 assessments conducted which included 15,264 assessment onshore over a 5 year period and 6439 assessments offshore over a 3 year period. The mean overall K10 score onshore was 18.85, while offshore it was 24.37. K10 scores increased with length of time detained both onshore and offshore, with K10 scores offshore generally higher at each time point. The results of this study add to a growing body of evidence that suggests that length of time detained and particularly offshore detention has a substantial impact on mental health.

## Introduction

Australia has maintained a policy of mandatory immigration detention for almost three decades. While this policy has been controversial for a range of reasons, the impact that detention has on health and wellbeing has been a persistent complaint, criticised domestically and internationally. Over the last 8 years, these concerns have become increasingly pressing as Australia re-opened offshore centres on Manus Island (Papua New Guinea) and Nauru. These centres have witnessed multiple riots, violence, sexual and physical abuse, self-harm and suicides. While onshore detention has been widely criticised, there is substantial anecdotal evidence to suggest offshore immigration detention is far more damaging and has a more serious impact on health and wellbeing [1].

While the Australian government has long withheld data that would give greater insight into these issues, evidence has emerged from a range of sources. Evidence suggests that detention has a devastating impact on health, with exceptionally high rates of mental distress and disorder [2–6] and that levels of distress often increase with length of time detained [7–9]. At least two studies have utilised the Kessler 10 (K10; see below) to measure psychological distress. Young and Gordon [9] reported on K10 results from a sample of 1,384 detainees held in onshore detention, between February and June 2014. The overall mean K10 score was 16.64 with females scoring significantly higher than males. The mean time in detention for those who completed the K10 was close to 50 weeks. They estimated that mean K10 score increased 0.036 points per week of detention. Mares [10] examined K10 scores amongst a sample of 166 refugees detained on Christmas Island. She concluded that the K10 indicated severe co-morbid depression and anxiety in 83% of adults and 85.7% of teenagers. Those in this sample were detained for 62 days on average. Similar concerns have been raised about offshore detention, with frequent reports of violence, riots, abuse, self-harm and suicidal behaviour [11]. While empirical evidence remains limited, over the last few years a number of reports have emerged. For example, the Médecins Sans Frontières [12] (MSF) Indefinite Despair Report found that amongst the 208 refugee and asylum seekers they had assessed on Nauru 129 (62%) were diagnosed with moderate to severe depression. The second highest diagnosis was anxiety disorder (25%), followed by PTSD (18%), mild depression (11%), complex trauma (6%) and resignation syndrome (6%), also known as traumatic withdrawal syndrome (for more, please see Newman et al. [13]). For the 74 refugees and asylum seekers seen over time, 15 (20%) remained stable, while 51 (69%) deteriorated and only 8 (11%) showed improvement in their daily functioning. Hedrick, Armstrong, Coffey and Borschmann [14] utilised health records to analyse episodes of self-harm between August 2014 and July 2015, comparing this against the average estimated adult population figures for that period. There were 949 self-harm episodes reported in total. Rates of self-harm ranged from 5 per 1000 asylum seekers in community-based arrangements to 260 per 1000 asylum seekers in offshore detention on Nauru. Rates were highest among asylum seekers in offshore and onshore detention facilities, and lowest among asylum seekers in community-based arrangements and community detention. As a comparison, rates of self-harm in the Australian community between 2012 and 2013 were 1.2 per 1000 people, meaning rates of self-harm in onshore and offshore detention were up to 216 times higher.

### Study Aims

Despite this growing evidence, there are no studies that have directly compared onshore and offshore immigration detention in Australia, nor elsewhere in the world. Furthermore, there are few studies that have examined the impact of immigration detention over time, utilising validated psychometric instruments. This study seeks to explore 4 years of K10 data from onshore and offshore Australian immigration detention centres from 2014 to 2018, examining the impact that the length and location of detention had on K10 scores, with comparisons between onshore and offshore detention centres. The dataset analysed here is the largest sample we know of in any research that has been conducted within Australian immigration detention centres (and perhaps to our knowledge, one of the largest globally) and the first to utilise a validated scale which offers a direct comparison between onshore and offshore detention.

## Methods

### Data Sources

In this study we utilised the Australian government’s Quarterly Immigration Detention Health Reports from 2013 to 2018. Two reports are provided each quarter, one on onshore immigration detention centres, that is, every detention centre in Australian and Christmas Island, while the other reports on offshore immigration detention centres, that is, centres on Manus and Nauru. Data in these reports is routinely collected by the immigration detention healthcare provider, International Health and Medical Services and reported to the Australian government. These reports contain a range of data about the health and wellbeing of detainees, including diagnoses, number of appointments and hospitalisations, among other variables. These reports were either already publicly available or obtained through Freedom of Information Requests sent to the Australian Department of Home Affairs. Under Australian law, Freedom of Information laws give everyone the right to access copies of documents held by the Australian government (or its agencies).

### Design and Participants

In both onshore and offshore detention centres, detainees are offered an assessment upon arrival and then at six month intervals. After they have been detained for 18 months, these assessments are offered every three months. The K10 was one of a number of assessment tools used, however these other assessments are not reported consistently in the quarterly health reports. The above data left us with what could be best described as a repeated measures cross sectional design. That is, while some participants would have been followed over time, many more were likely to have completed K10 assessments intermittently and repeatedly at different points in time. In particular those detained offshore were likely to have completed an assessment multiple times. To better understand this, it is worth noting the differences in detention population. The population in onshore immigration detention centres has been generally more diverse and dynamic than the offshore population. While onshore detention houses refugees and asylum seekers, the majority of those detained have either overstayed their visa or had it cancelled. Also, while many people are still detained for protracted periods, a substantial number is only detained for short periods of time, prior to deportation for example. During 2012–13 about 40,000 people were held in onshore detention, however today and since the cessation of boat arrivals, this number generally sits between 5 and 10,000 annually [15].

The offshore immigration detention population differs substantially, with every detainee either an asylum seeker or refugee who travelled to Australia by boat in 2013 or 2014. All who arrived in Australia in this way were transferred offshore, with the government vowing to never to resettle them in Australia [16]. Throughout 2014, boat arrivals almost ceased completely, leaving thousands indefinitely detained on Manus and Nauru. Since these policies were introduced, 4177 people have been detained offshore; today hundreds remain on Manus Island and Nauru awaiting resettlement [15].

### Kessler 10

The Kessler-10 (K10) is a self-report measure of psychological distress based on questions about levels of anxiety and depression over the last four weeks [17]. Each item is scored from 1 to 5, from “none of the time”—“all of the time”. The total score is the sum of all 10 items 1–10. Scores range from 10 to 50 with higher scores indicating greater distress. Generally, those scoring under 20 are likely to be well, those who score 20–24 are likely to have symptoms of a mild mental disorder, those who score 25–29 are likely to have moderate symptoms and those who score 30 and over are likely to have severe. Higher scores are also more predictive of being diagnosed with mental illness symptoms [18]. The K10 has been validated in population-based and clinical populations and with a wide range of language and cultural groups, including refugee populations [19, 20].

In this data set K10 scores were recorded for onshore and offshore centres, by length of detention at five intervals (0–3 months, 4–6 months, 7–12 months, 13–18 months and 19+ months). Additionally, K10 scores were also reported by number of participants who fell into each category above, that is, low (scoring < 20), mild (scoring 20–24), moderate (scoring 25–29) and severe (scoring 30+). Onshore data was available from Q1 2014–Q4 2018 (20 quarters). Offshore data was available from Q2 2014–Q2 2017 (13 quarters). Manus and Nauru data were available from Q3 2015–Q2 2017 (8 quarters).

### Ethical Approval

Ethics approval was granted by the University of Greenwich, Human Research Ethics Committee (UREC/20.1.5.6).

### Analysis

Data was manually entered from the quarterly health reports and screened by two authors (RE and EK). A number of issues were noted where data was recorded inconsistently in the quarterly reports. Where possible these were recalculated by the authors, if this was not possible data was excluded from analysis. Descriptive statistics were calculated. No further analysis was conducted as this data violated assumptions of independence of observations. That is, it is likely that the same individuals appeared multiple times across quarters both onshore and offshore; this limited the analyses we could conduct and comparisons we could make between onshore and offshore data.

## Results

Over a 5 year period there were 21,703 K10 assessments conducted which included 15,264 assessments onshore over a 5 year period and 6439 assessments offshore over a 3 year period. There were no assessments on Manus and Nauru for those detained between 0–3 and 4–6 months as Manus and Nauru data was reported separately until Q3 2015, by which time all detainees had already been detained for over 12 months. Descriptive results along with the spread of K10 scores are reported in Table 1. K10 scores increased substantially over time, with mean scores of 15.01 recorded onshore at 0-3 months, compared to scores of 21.91 reported at 19+ months. Scores for those offshore also follow a similar pattern, increasing with length of detention, however scores offshore are also substantially higher, for example, those detained offshore for 19+ months (both on Manus Island and Nauru) had a mean K10 score of 25.62. Overall, across all time periods offshore mean K10 scores were higher (24.37) than for those detained onshore (18.85).

**Table 1:** Number of assessments and K10 mean scores

Length of time detained	Onshore		Offshore		Manus		Nauru	
	n	Mean (SD)	n	Mean (SD)	n	Mean (SD)	n	Mean (SD)
0–3	4709	15.01 (0.81)	151	29.05 (4.72)	–	–	–	–
4–6	1165	19.44 (2.04)	164	19.06 (3.30)	–	–	–	–
7–12	2492	19.73 (2.17)	1199	18.47 (4.06)	–	–	4	15.67 (5.18)
13–18	2189	20.14 (2.71)	1676	23.14 (5.20)	7	23.34 (1.89)	133	25.49 (6.09)
19+	4709	21.91 (1.61)	3303	25.62 (3.65)	1362	25.23 (1.92)	796	27.66 (4.80)
Total	15,264	18.85 (1.52)	6439	24.37 (3.79)	1369	25.17 (1.83)	933	27.68 (4.74)

Onshore data was available from Q1 2014–Q4 2018. Offshore data was available from Q2 2014–Q2 2017. Manus and Nauru data were available from Q3 2015–Q2 2017

**Fig. 1:**
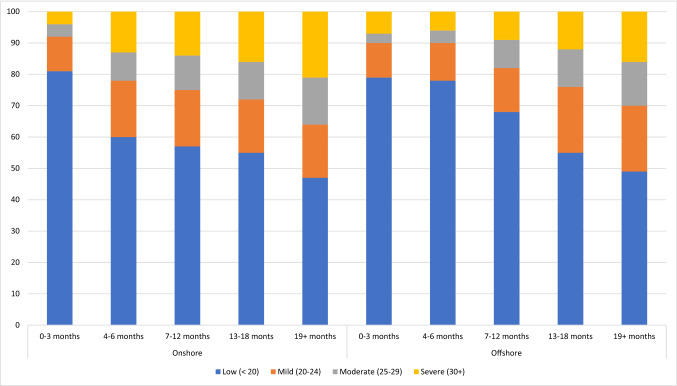
Percentage of detainees who scored in each K10 category, onshore and offshore

**Fig. 2:**
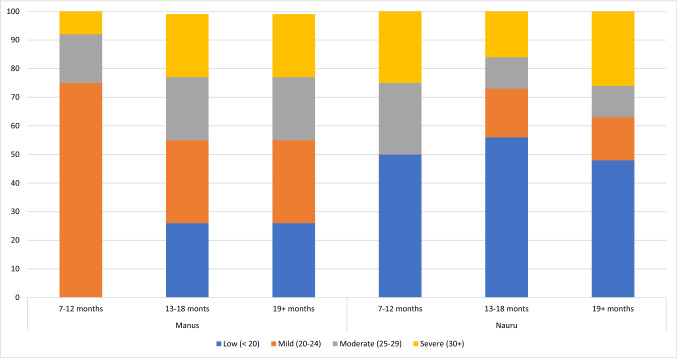
Percentage of detainees who scored in each K10 category, Manus Island and Nauru

In addition to mean scores, the number of detainees who fall into each K10 category were reported each quarter. These results were collated and are reported in Table 2. Results here show a similar trend, namely that in the groups where individuals had been detained the longest, far more detainees were likely to score in the higher K10 categories. Both onshore and offshore, almost 50% of people detained 13–18 and 19+ months scored 20+ on the K10, with over 20% of those detained 19+ months scoring 30+ . A similar picture is seen when looking at each offshore centre separately, in that as length of detention increased, more and more people generally fell into higher K10 categories, however on Manus, for those detained 7–12, 13–18 and 19+ months, about 75% of detainees scored 20+ . These results are summarised in Table 2 and Figs. 1 and 2.

**Table 2:** Number and percentage of scores falling in each K10 band by length of detention

	Low (< 20)	Mild (20–24)	Moderate (25–29)	Severe (30+)	Total
Onshore					
0–3	3790 (81%)	526 (11%)	196 (4%)	197 (4%)	4709
4–6	665 (60%)	207 (18%)	113 (9%)	180 (13%)	1165
7–12	1494 (57%)	403 (18%)	240 (11%)	355 (14%)	2492
13–18	1248 (55%)	305 (17%)	272 (12%)	364 (16%)	2189
19+	2152 (47%)	812 (17%)	798 (15%)	947 (21%)	4709
Total	9349 (62%)	2253 (15%)	1619 (10%)	2043 (13%)	15,264
Offshore					
0–3	120 (79%)	16 (11%)	4 (3%)	11 (7%)	151
4–6	126 (78%)	20 (12%)	7 (4%)	11 (6%)	164
7–12	810 (68%)	164 (14%)	108 (9%)	117 (9%)	1199
13–18	910 (54%)	353 (21%)	208 (12%)	205 (12%)	1676
19+	1265 (38%)	789 (24%)	564 (17%)	691 (21%)	3303
Total	3227 (49%)	1341 (21%)	890 (14%)	1032 (16%)	6493
Manus					
0–3	–	–	–	–	–
4–6	–	–	–	–	–
7–12	–	–	–	–	–
13–18	0 (0%)	4 (75%)	2 (17%)	1 (8%)	7
19+	356 (26%)	396 (29%)	304 (22%)	306 (22%)	1362
Total	356 (26%)	400 (29%)	306 (22%)	307 (22%)	1369
Nauru					
0–3	–	–	–	–	–
4–6	–	–	–	–	–
7–12	2 (50%)	0 (0%)	1 (25%)	1 (25%)	4
13–18	75 (56%)	23 (17%)	14 (11%)	21 (16%)	133
19+	379 (48%)	118 (15%)	85 (11%)	214 (27%)	796
Total	456	141	100	326	933

## Discussion

This study sought to examine the impact of length and location of detention on K10 scores amongst those detained in Australian onshore and offshore immigration detention. Our results suggest that psychological distress increased with length of detention, with those detained offshore more likely to report higher levels of psychological distress compared to those detained onshore. These findings are consistent with existing empirical and anecdotal evidence that not only suggest that length of detention increases psychological distress, but that this is also exacerbated by being detained offshore. A particularly striking observation from our data is the degree to which K10 scores increase after being detained for 3 months. Our results suggest that those detained for less than three months scored similarly to an Australian community sample on the K10 (see below), while detention beyond this time resulted in significantly greater psychological distress. One result that appears to be an outlier is also worth discussing here. Those detained offshore for 0–3 months scored relatively high on the K10, with a mean score of 29.05 amongst 151 detainees. These high scores are readily explained. Almost every score in this group was captured between March–June 2014. This was the first quarter where data was available offshore and it was not long after the Australian government had announced its policy of offshore processing and a policy of not resettling those who travelled by boat in Australia. At this time many were still being forcefully transferred from Australian territories to Manus Island and Nauru. Furthermore, on Manus Island in February 2014 there was a riot where over 70 detainees were injured and one was killed, with centre guards later convicted of murder [21]. The elevated scores seen amongst those in the offshore group who had been detained for 0–3 months likely reflected these events.

Given the nature of this data some comparisons are also warranted with Australian community K10 scores. Slade, Grove and Burgess [22] examined K10 scores amongst 8,841 participants from an Australian community sample. The mean K10 score was 14.05. Amongst those with diagnosed mental health conditions, mean scores were generally higher; those with affective disorders had a mean score of 23.2, those with anxiety disorders a mean score of 19.8 and those with substance abuse disorders had a mean score of 18. Of those who scored 22–29, 57% were diagnosed with mental illness over a 12 month period. Those who scored 30–50, had an almost 80% chance of being diagnosed with a mental illness over a 12 month period. The results reported in this study, on average are much higher and more closely resemble the mean scores of those in the Australian community diagnosed mental health conditions. The results here are also higher than that reported in refugees in the Australian community. Lillee, Thambiran and Laugharne [23] examined K10 scores amongst newly resettled refugees in Western Australia. Amongst 300 participants, K10 scores ranged from 14 for refugees from South-East Asia to 19 for those from Western/Southern Asia. Again, the scores of this sample and particularly those who were detained for longer than 3 months, were either similar or higher. Finally, the scores reported here are also higher than those reported by Young and Gordon [9] who reported mean K10 scores of 16.64, amongst detainees who had be held for almost 12 months. Like our results, this study also observed that K10 scores increased with time detained. Finally, it should be noted that the results reported here are generally consistent with other information contained in the quarterly health reports, namely that health needs are high amongst onshore and offshore detained, however those detained offshore generally have higher health needs [24].

While our results suggest that length of detention and location have an impact on psychological distress as measured by the K10 caution is warranted. Because of limitations with the data reported in the governments quarterly health reports, we were only able to present descriptive statistics. Not only can we not assume a cause and effect relationship, caution is warranted more generally, we cannot say comprehensively whether there was a statistically significant change in K10 scores with length of time detained and location. Second, in comparing onshore and offshore populations it should be kept in mind that these populations are not directly comparable, that is, we cannot rule out that the higher scores seen offshore are at least partly because of the differences in population characteristics. Third, we can only speculate, but in particular with the offshore population there was likely an under-reporting of psychological distress and therefore lower K10 scores reported. Multiple quarterly health reports from which we extracted data noted that there were issues in engaging with those most distressed and warned that levels of distress may have been underreported. In short, care is needed as it cannot be assumed that the K10 scores reported are representative of the entire detention population, nor are applicable to other detention settings. On this point and finally in regards to generalisability, while Australian immigration detention may have features similar to other countries, the conditions within centres vary greatly and policy has evolved over time. Similar limitations were noted by von Werthen et al. [4] in a systematic review on the impact of immigration detention on mental health; notably many studies failed to provide adequate detail about the conditions of detention, limiting the conclusions that could be drawn regarding generalisability.

These results have several implications for policy. First, while these results suggest that those detained offshore had far worse mental health outcomes than detainees onshore, they say little about reform or what could be done to improve care offshore. However, taken together with other evidence, these results bolster calls for an end to offshore asylum processing. That is, along with creating far worse health outcomes, the broader literature suggests little way to address health and wellbeing while current policies remain in place [25]. To date, the Australian government has spent billions on healthcare offshore [26], however, as long as the purpose of detention is to deter others from coming to Australia, these issues will remain, regardless of the healthcare services available [27]. Additionally, there are compelling human rights reasons to abolish offshore detention, with such policies being identified as cruel and degrading [28] and with several domestic and international human rights bodies calling for the abolition of offshore detention [29]. These results also support calls for the abolition (or at the very least, significant reform) of onshore immigration detention [30] and the delivery of healthcare onshore [31]. Finally, while we have called for caution in relation to the generalisability of these results, several conclusions can still be drawn. At this point in time a number of countries seeking to emulate these policies [32]. Our results should serve as a warning for other countries that are looking to model Australia’s approach to immigration detention and, in particular, offshore immigration detention.
